# The Development and Evaluation of a Retrieval-Augmented Generation Large Language Model Virtual Assistant for Postoperative Instructions

**DOI:** 10.3390/bioengineering12111219

**Published:** 2025-11-07

**Authors:** Syed Ali Haider, Srinivasagam Prabha, Cesar Abraham Gomez Cabello, Ariana Genovese, Bernardo Collaco, Nadia Wood, James London, Sanjay Bagaria, Cui Tao, Antonio Jorge Forte

**Affiliations:** 1 Division of Plastic Surgery, Mayo Clinic, Jacksonville, FL 32224, USA; 2 Department of Radiology AI IT, Mayo Clinic, Rochester, MN 55905, USA; 3 Department of Surgery, Mayo Clinic, Jacksonville, FL 32224, USA; 4 Division of Surgical Oncology, Mayo Clinic, Jacksonville, FL 32224, USA; 5 Department of Artificial Intelligence and Informatics, Mayo Clinic, Jacksonville, FL 32224, USA; 6 Center for Digital Health, Mayo Clinic, Rochester, MN 55905, USA

**Keywords:** artificial intelligence, retrieval augmented generation (RAG), virtual assistant, large language models (LLM), healthcare chatbot, post-operative care, patient education

## Abstract

Background: During postoperative recovery, patients and their caregivers often lack crucial information, leading to numerous repetitive inquiries that burden healthcare providers. Traditional discharge materials, including paper handouts and patient portals, are often static, overwhelming, or underutilized, leading to patient overwhelm and contributing to unnecessary ER visits and overall healthcare overutilization. Conversational chatbots offer a solution, but Natural Language Processing (NLP) systems are often inflexible and limited in understanding, while powerful Large Language Models (LLMs) are prone to generating “hallucinations”. Objective: To combine the deterministic framework of traditional NLP with the probabilistic capabilities of LLMs, we developed the AI Virtual Assistant (AIVA) Platform. This system utilizes a retrieval-augmented generation (RAG) architecture, integrating Gemini 2.0 Flash with a medically verified knowledge base via Google Vertex AI, to safely deliver dynamic, patient-facing postoperative guidance grounded in validated clinical content. Methods: The AIVA Platform was evaluated through 750 simulated patient interactions derived from 250 unique postoperative queries across 20 high-frequency recovery domains. Three blinded physician reviewers assessed formal system performance, evaluating classification metrics (accuracy, precision, recall, F1-score), relevance (SSI Index), completeness, and consistency (5-point Likert scale). Safety guardrails were tested with 120 out-of-scope queries and 30 emergency escalation scenarios. Additionally, groundedness, fluency, and readability were assessed using automated LLM metrics. Results: The system achieved 98.4% classification accuracy (precision 1.0, recall 0.98, F1-score 0.9899). Physician reviews showed high completeness (4.83/5), consistency (4.49/5), and relevance (SSI Index 2.68/3). Safety guardrails successfully identified 100% of out-of-scope and escalation scenarios. Groundedness evaluations demonstrated strong context precision (0.951), recall (0.910), and faithfulness (0.956), with 95.6% verification agreement. While fluency and semantic alignment were high (BERTScore F1 0.9013, ROUGE-1 0.8377), readability was 11th-grade level (Flesch–Kincaid 46.34). Conclusion: The simulated testing demonstrated strong technical accuracy, safety, and clinical relevance in simulated postoperative care. Its architecture effectively balances flexibility and safety, addressing key limitations of standalone NLP and LLMs. While readability remains a challenge, these findings establish a solid foundation, demonstrating readiness for clinical trials and real-world testing within surgical care pathways.

## 1. Introduction

### 1.1. Background

Each year, more than 313 million surgical procedures are performed worldwide [1], including approximately 50 million in the United States [2]. Rising surgical volumes, coupled with the shift toward same-day or short-stay procedures [3], have increasingly transferred the responsibility for recovery from hospitals to patients and their caregivers [4]. Many patients are discharged while still under the residual effects of anesthesia or analgesics, limiting their ability to absorb essential postoperative instructions [5]. Families and caregivers, who often lack formal medical training, are frequently required to assume the role of primary care coordinators [6].

This transition creates significant challenges, given variable health literacy [7,8], chronic comorbidities, and limited support systems [8]. Cognitive barriers further complicate recovery [9], as patients typically forget more than half of verbal instructions provided at discharge [10]. Unresolved questions often lead to high volumes of non-urgent clinic calls, electronic health record (EHR) messages, and unnecessary visits [11]. These repetitive interactions strain provider resources, reduce efficiency, and divert attention from higher-acuity care [12]. Over time, this cycle of patient uncertainty and repeated inquiries contributes to cognitive overload [13], burnout [14], and diminished satisfaction among surgical teams.

### 1.2. Limitations of Current Patient Education Methods

Because discharge materials are often insufficient, follow-up interactions frequently become the primary mode of patient education [15]. This dependence reflects the shortcomings of standard resources, including written handouts and patient portals, which are static, text-heavy, and often unengaging [16,17]. Patients frequently neglect or misplace these materials [18], and when they do use them, the content is commonly presented in complex medical jargon that exceeds average health literacy levels [19]. Importantly, these resources do not permit interactive questioning, leaving patients unable to clarify uncertainties or explore information in a way that promotes true understanding.

Beyond limited comprehension, these resources also lack adaptability. They cannot adjust to a patient’s specific procedure, stage of recovery, or evolving concerns, resulting in generic guidance that fails to meet diverse needs. This lack of engagement, personalization, and interactivity disproportionately affects vulnerable populations, exacerbating health disparities. Ultimately, these information gaps increase the risk of preventable complications such as medication errors [20], improper wound care [20], and delayed recognition of warning signs [21]. Since well-informed patients make better health decisions, optimizing discharge education is critical to enhancing patient safety, improving outcomes, and strengthening hospital quality metrics. Table 1 summarizes the major limitations of current patient education resources, their impact on patients and providers, and the specific gaps addressed by the AIVA Platform.

### 1.3. The Promise of AI in Postoperative Care

Artificial intelligence (AI) offers scalable solutions to bridge these persistent gaps in postoperative education [22]. With rising surgical volumes and limited clinical resources, AI systems can deliver consistent, real-time support beyond the walls of the hospital [23,24]. Unlike static handouts or portals, conversational virtual assistants allow patients to ask natural language questions and receive immediate, context-specific answers, closely simulating the interaction of a clinical encounter.

Interactivity is a key advantage of AI-powered virtual assistants. By enabling question-and-answer exchanges, these tools help patients clarify uncertainties and better understand instructions, reducing reliance on follow-up calls or visits. They also provide continuous availability, offering patients 24/7 access to accurate information [25]. Crucially, virtual assistants can personalize guidance according to the type of surgery [26], stage of recovery [26], and specific patient concerns [27], delivering dynamic support that adapts to evolving needs [28].

Beyond surgery, AI-driven virtual assistants have already shown feasibility and effectiveness across a range of healthcare applications, including chronic disease management [29], medication adherence [30], mental health support [31], and administrative tasks [31]. Patients are increasingly willing to trust conversational agents for basic health information, with evidence suggesting that large language model (LLM)-based assistants can deliver accurate and reliable responses even for complex information needs [32]. These developments highlight both the potential and the risks of conversational AI in healthcare, which directly informed the design of our own virtual assistant.

### 1.4. The Evolution of AI Virtual Assistant

Building on this emerging potential, our team began exploratory development of the AI Virtual Assistant (AIVA) in 2018 using IBM Watson Assistant. In an initial pilot study (July–August 2018), AIVA was trained on ten common preoperative plastic surgery topics with standardized responses, followed by iterative testing to improve recognition of diverse patient phrasings. For subsequent validation, including a patient satisfaction study conducted between December 2020 and February 2021, the system was migrated to Google Dialogflow. The outcomes of these stages were later published, reporting 93–98% accuracy for frequently asked postoperative questions [33] and high patient satisfaction (88%) [34].

However, early systems were constrained by their reliance on scripted, intent-based responses. While accurate for narrowly defined questions, they struggled with variations in phrasing, ambiguity, or unexpected queries due to their shallow semantic understanding. At the same time, standalone LLMs such as ChatGPT and Gemini have shown remarkable fluency and adaptability but carry risks of hallucinations, inaccuracies, and biases that limit their safe use in clinical care [35]. A comparative study confirmed that intent-based NLP systems outperformed commercial LLMs in safety and accuracy for surgical FAQs, underscoring the need for a hybrid approach [36].

Retrieval-Augmented Generation (RAG) architectures have since emerged as a promising solution, combining the flexibility of LLMs with the reliability of curated knowledge bases [37]. By grounding generative outputs in validated clinical content, RAG offers an effective pathway to deliver conversational fluency without compromising medical safety—making it particularly well-suited for postoperative education [38,39].

### 1.5. Study Objectives

Emerging safety frameworks emphasize the need for guardrails [40], transparency [41], and rigorous validation in clinical AI systems [40]. Foundational principles, such as explainability, predictability, and controllability, are essential prerequisites to minimize hallucinations and ensure evidence-grounded outputs [42]. Given these critical challenges, this study aimed to develop and evaluate a RAG-LLM Virtual Assistant called ‘The AIVA Platform’ for postoperative care, built on Google’s Vertex AI platform [43]. Its performance was rigorously assessed using a synthetic corpus of patient queries, with evaluation including both expert reviews and automated metrics [44].

## 2. Methods

### 2.1. Study Design

**Figure 1:**
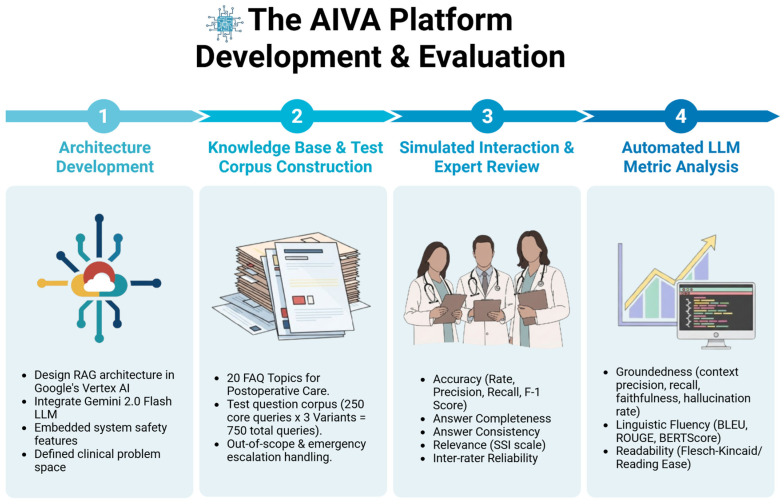
The AIVA Platform’s development and evaluation workflow. The process was organized into four stages: (1) Architecture Development—design of the retrieval-augmented generation (RAG) system in Google’s Vertex AI, integration of Gemini 2.0 Flash LLM, and implementation of embedded safety features; (2) Knowledge Base and Test Corpus Construction—creation of a curated, physician-verified knowledge base across 20 high-frequency postoperative topics and development of a 750-query test corpus including out-of-scope and emergency scenarios; (3) Simulated Interaction and Expert Review—blinded physician reviewers evaluated accuracy, completeness, consistency, and relevance, with inter-rater reliability testing; and (4) Automated LLM Metric Analysis—performance was further assessed with metrics for groundedness (context precision, recall, faithfulness), fluency (BLEU, ROUGE, BERTScore), and readability (Flesch–Kincaid/Reading Ease). The AIVA Platform’s Development and Evaluation Workflow, illustrating key stages from architecture design to automated metric analysis. Created in BioRender. Haider, S. (2025) https://BioRender.com/fg43djc.

We developed the AIVA Platform on Google’s Vertex AI (Google LLC, Mountain View, CA, USA), leveraging its comprehensive capabilities. We chose this platform for its robust scalability, high performance, and seamless integration with other Google Cloud services, complemented by strong security and user-friendly tools. We then evaluated the AIVA Platform using systematically generated, simulated patient queries. This approach allowed a comprehensive, controlled assessment across diverse postoperative care scenarios without exposing live patients to safety risks. This also enabled the structured exploration of rare, safety-critical scenarios, such as emergency escalations or out-of-scope queries, which can occur unpredictably in real-world clinical practice. Our rigorous evaluation included both expert human quality ratings (for accuracy, completeness, consistency, and relevance) and automated LLM metrics (for groundedness, fluency, readability, and linguistic characteristics). As shown in Figure 1, the workflow progresses from knowledge base development to architecture design, system deployment, and finally evaluation using both physician reviewers and automated metrics.

### 2.2. Knowledge Base Development

We developed the knowledge base through an iterative process, ultimately expanding it to encompass 20 high-frequency postoperative topics, reflecting expert clinical consensus and continuous refinement. Topics were selected based on their relevance to common patient questions, clinical significance, and areas of frequent patient confusion. Physicians curated content exclusively from current clinical practice guidelines (such as American College of Surgeons (ACS) Enhanced Recovery After Surgery (ERAS) guidelines), institutional protocols (CDC surgical-site infection prevention recommendations, WHO Safe Surgery protocols), patient education materials, and peer-reviewed literature. All knowledge was formatted to patient-friendly handouts for ingestion into the retrieval pipeline. Table 2 details the 20 comprehensive postoperative topics included in the knowledge base.

### 2.3. System Architecture

**Figure 2:**
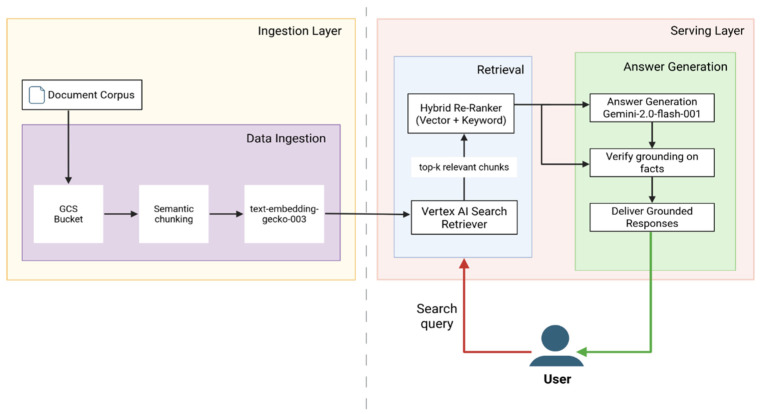
Technical architecture of the AIVA Platform based on Google Vertex AI. The system consists of two main layers: the Ingestion Layer (left) and the Serving Layer (right). In the Ingestion Layer, clinical documents are securely uploaded to a Google Cloud Storage (GCS) bucket, processed using semantic chunking, and converted into embeddings with the text-embedding-gecko-003 model for indexing. In the Serving Layer, patient queries are processed through a Retrieval Path and an Answer Generation Path. The Retrieval Path uses the Vertex AI Search retriever with hybrid re-ranking (vector similarity and keyword-based BM25) to identify the top relevant chunks from the knowledge base. These evidence passages are then passed to the Answer Generation Path, where Gemini 2.0 Flash-001 produces a draft response. Each response undergoes grounding verification against the retrieved evidence and heuristic safety checks before delivering a final, clinically verified answer to the user. Created in BioRender. Haider, S. (2025) https://BioRender.com/7s0m5ii.

The AIVA Platform, powered by the Gemini 2.0 Flash-001 LLM, was deployed on Google Vertex AI. Its robust architecture is organized into two main layers: the Ingestion Layer, which prepares the clinical knowledge base, and the Serving Layer, which processes user queries and generates verified responses. The pipeline effectively ingests and transforms clinical documents, then retrieves the most relevant evidence, re-ranks it, and feeds it to the LLM under strict grounding and safety gates. Figure 2 illustrates this technical workflow, beginning with ingestion of clinical documents (left panel), followed by retrieval and re-ranking (center), and ending with grounded, patient-facing responses with embedded safety checks (right panel). Currently, no formal technical standards exist for RAG workflows in healthcare. The architecture shown in Figure 2 therefore represents a novel engineering approach. To ensure clinical relevance, the knowledge base was built from authoritative guidelines such as ACS ERAS protocols, CDC infection-prevention recommendations, and WHO Safe Surgery standards, ensuring that system outputs remain aligned with established national and global postoperative care practices. This architecture provides fast, verified responses, making it ideal for reliable postoperative patient guidance.

#### 2.3.1. Ingestion Layer—Knowledge Base Preparation

The Ingestion Layer is responsible for securely processing and preparing clinical source documents, including patient-education materials, postoperative discharge instructions, and medication protocols. All content is securely uploaded to a private Google Cloud Storage (GCS) bucket, protected by Virtual Private Cloud (VPC) Service Controls. Vertex AI Search then initiates an automated ingestion process. This process first involves semantic chunking, where the Vertex AI layout-aware parser segments documents into concise, semantically coherent passages (approximately 500 characters each, without overlap) to ensure unique representation and improved retrieval precision. Non-overlapping segments were selected to avoid redundant embeddings and prevent diluted retrieval precision that occurs when overlapping windows introduce repeated content across chunks. Subsequently, for embedding generation, each text passage is converted into a dense 768-dimensional vector embedding using Google’s multilingual textembedding-gecko-003 model. This preprocessing helps avoid latency delays during real-time interactions. Embeddings are stored in a hierarchical navigable small-world (HNSW) approximate nearest-neighbor index (M = 32, efConstruction = 200) for scalable, low-latency retrieval.

#### 2.3.2. Serving Layer—User Query Processing and Response Generation

When a patient submits a query, the Serving Layer activates, orchestrated by the Vertex AI Agent Builder Playbook using the Reasoning and Action (ReAct) paradigm. This layer consists of two interconnected paths: Retrieval and Answer Generation.

##### Retrieval Path

This path is responsible for identifying the most relevant information from the knowledge base. Embeddings and essential metadata are stored in a fully managed Vertex AI Search index, leveraging an Approximate Nearest Neighbor (ANN) structure that ensures scalability, high availability, and rapid query performance. At runtime, the Playbook embeds the user’s question using the textembedding-gecko-003 model and queries this ANN-based index, retrieving the ten passages most similar to the query embedding. These initial ten candidate passages then undergo hybrid re-ranking, a process combining vector-based cosine similarity with BM25 keyword relevance, which prioritizes passages containing important yet infrequently occurring clinical terms. Following re-ranking, the top three passages constitute the final evidence set passed to the language model for answer generation. Three passages were selected as optimal based on tuning experiments showing this provided the best balance between answer completeness and response brevity within Gemini 2.0 Flash’s context window limitations.

##### Answer Generation Path

This path processes the retrieved information to produce the patient-facing response. The Agent Builder Playbook first dynamically constructs an augmented prompt, combining a predefined role instruction (“You are a postoperative-care assistant”), the patient’s question, and the three evidence passages retrieved; this prompt ensures the language model operates within its context limit. This augmented prompt is then sent to Gemini 2.0 Flash-001 for draft response generation. The Gemini-generated draft subsequently undergoes validation via a grounding verification process, evaluating each sentence for semantic alignment with the evidence passages and accepting only responses where all sentences demonstrate high grounding confidence. A heuristic validation step further screens for fabricated numerical values or unsupported references. Upon successfully passing both grounding and heuristic checks, the final response is transmitted to the chatbot’s user interface; evidence snippets are preserved internally for auditing purposes.

#### 2.3.3. Embedded Safety Features

Beyond the core retrieval and generation pipeline, the AIVA Platform integrates robust safety features throughout its architecture. These encompass automated out-of-scope filtering for non-medical queries, emergency escalation detection protocols, and predefined safety handoff responses for cases identified as clinically high risk.

### 2.4. Test Question Corpus

We systematically generated 750 unique simulated patient queries to evaluate the AIVA Platform. This comprehensive test corpus was designed to reflect anticipated real-world interactions and balanced clinical coverage, linguistic diversity, and safety-critical edge cases. It included 600 in-scope clinical queries, derived from 200 core questions (each paraphrased into two distinct variants); 120 out-of-scope non-medical queries, derived from 40 base queries, designed to assess safety; and 30 emergency escalation scenarios, derived from ten base scenarios, to test critical safety responses (e.g., dyspnea, uncontrolled bleeding). The full composition of the test corpus is summarized in Table 3.

### 2.5. Evaluation Procedures

According to Abbasian et al., effective evaluation of healthcare chatbots requires a comprehensive, multi-metric approach [44]. Traditional generic or intrinsic (automated) LLM metrics often fail to capture essential medical nuances or crucial user-centered aspects like empathy and trust. Similarly, extrinsic (human-based) evaluation methods, while incorporating human judgment, have historically overlooked key elements such as emotional support and personalization. Consequently, our evaluation integrated both automated classification and human expert review to ensure a thorough, user-centered assessment. The evaluation methodology employed different sample compositions for specific metrics to optimize statistical validity, as detailed in Table 4.

#### 2.5.1. Human Expert Evaluation

Gold standard reference answers were developed by two senior physicians based on current clinical guidelines, institutional discharge instructions, and peer-reviewed literature. These reference answers served as the benchmark for evaluating system responses.

**Figure 3:**
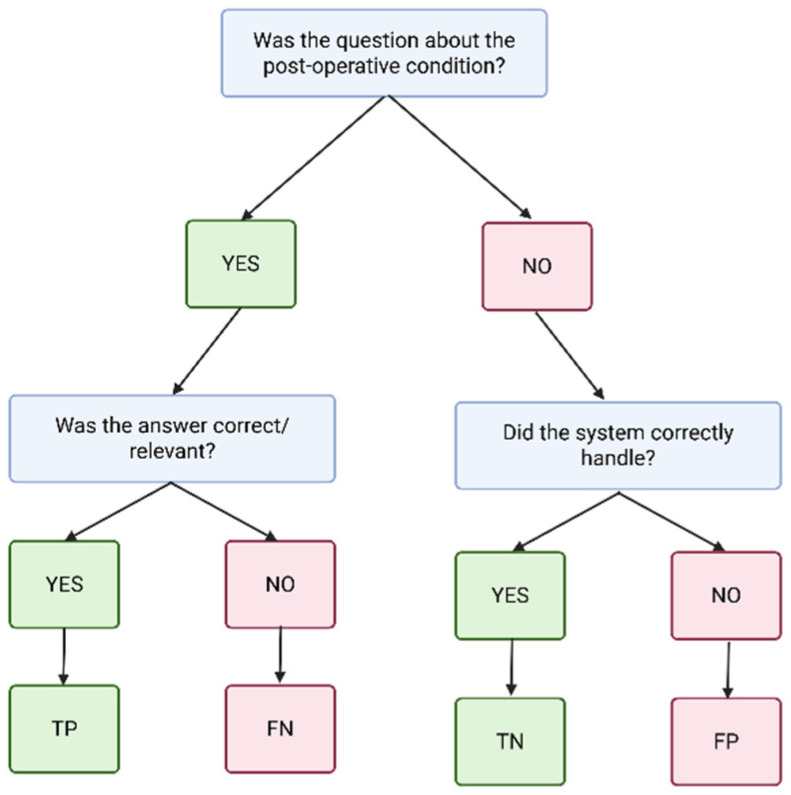
Accuracy classification flow diagram. Responses were categorized into four groups based on reviewer assessment: TP (true positive)—correct in-scope answers; FN (false negative)—in-scope queries where the system failed to provide the correct answer; TN (true negative)—correct rejections of out-of-scope queries; and FP (false positive)—inappropriate answers to out-of-scope queries. Created in BioRender. Haider, S. (2025) https://BioRender.com/jlewnbc.

Each system response was independently scored by three blinded physician reviewers. Disagreements were resolved using a majority-vote rule: if at least two reviewers rated a response as correct, the final classification was recorded as correct. This process enabled calculation of overall accuracy, precision, recall, and F1-score, as illustrated in Figure 3. Representative examples of queries, gold standard references, system outputs, and reviewer annotations are provided in Supplementary Table S1. These examples illustrate both straightforward cases with unanimous agreement and more complex cases where reviewer judgment differed, demonstrating how final consensus labels were determined.

Performance metrics were calculated using micro-averaging, which aggregates contributions from all queries across classes. This approach was chosen to ensure that each query contributed equally to the overall metrics, rather than allowing small classes (e.g., escalation scenarios) to disproportionately influence results.

Beyond accuracy, reviewers rated completeness and consistency of responses using a 5-point Likert scale, comparing system outputs to gold-standard reference answers. To assess relevance, we applied the Sensibility–Specificity–Interestingness (SSI) Index, which evaluates responses across three dependent dimensions on a 0–3 scale [45].

#### 2.5.2. Automated and Algorithmic Metrics

Beyond direct human expert scoring, we utilized a suite of automated and algorithmic metrics to assess broader system performance and linguistic characteristics.

Groundedness, which quantifies the hallucination rate, evaluated the factual validity and consistency of generated responses with provided evidence. The groundedness assessment involved a two-stage hybrid retriever [46], leveraging semantic (Vertex AI text-embedding-004 with FAISS/MMR on 300-word chunks) and lexical (BM25) searches to retrieve relevant knowledge base evidence. Groundedness quality was quantified using three fuzzy-Jaccard metrics: context precision (retrieved chunk support for gold passage), context recall (gold-passage sentences retrieved), and faithfulness (answer sentences supported by retrieved chunks) [47]. An LLM-as-judge check with Gemini 2.0-Flash provided secondary verification of answer-evidence consistency.

Linguistic fluency was evaluated using multiple natural language generation benchmarks, including BLEU, ROUGE-1, ROUGE-2, ROUGE-L, and BERTScore F1. Additional language structure metrics, such as total word count, sentence count, average sentence length, lexical diversity, and Flesch–Kincaid readability index, were also analyzed.

## 3. Results

The results are organized into two main themes. The first theme focuses on human expert evaluation, including classification accuracy, quality ratings (completeness, consistency, and relevance), inter-rater reliability, and safety outcomes. The second theme presents findings from automated large language model (LLM) metrics, which assess linguistic fluency, readability, and groundedness. Together, these complementary evaluations provide a comprehensive view of the AIVA Platform’s performance.

### 3.1. Human Expert Evaluation Results

#### 3.1.1. Accuracy (Classification)

**Figure 4:**
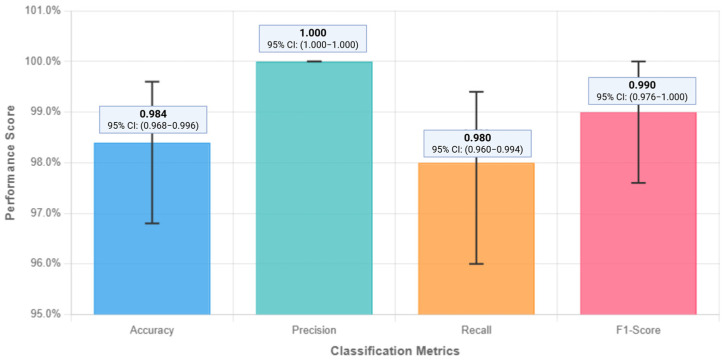
Classification metrics performance of the AIVA Platform. The system achieved an overall accuracy of 98.4%, with precision = 1.0, recall = 0.98, and F1-score = 0.9899. These results highlight the system’s ability to correctly identify in-scope queries while avoiding inappropriate responses to out-of-scope queries. Created in BioRender. Haider, S. (2025) https://BioRender.com/s52laj8.

The AIVA Platform demonstrated high classification accuracy across the 250-query core evaluation set. Of these, 196 were true positives (correct in-scope answers) and 50 were true negatives (correct out-of-scope rejections). No false positives occurred, and four responses were false negatives. This yielded an overall classification accuracy of 98.4% (95% CI: 96.8–99.6%) with precision 1.0, recall 0.98 (95% CI: 96.0–99.4%), and F1-score 0.9899 (95% CI: 97.6–100%). The margin of error was ±1.4%, demonstrating statistical precision adequate for proof-of-concept validation. Table 5 summarizes the full confusion matrix, and Figure 4 presents the classification metrics.

Per-topic performance analysis across 22 postoperative care domains showed consistent accuracy with minimal dispersion. Eighteen topics including out-of-scope and emergency escalation scenarios achieved 100% accuracy, while four topics—Physical Activity, Return to Work, Showering/Bathing, and Travel/Driving—each recorded one false negative, resulting in 90% accuracy. Mean topic-level accuracy was 98.2% ± 3.9%, with performance ranging from 90% to 100%. The four false negative cases clustered in activity-related queries involving timing and restriction guidance, suggesting these domains require more nuanced clinical decision-making frameworks.

#### 3.1.2. Human Quality Ratings (Completeness, Consistency, Relevance)

**Figure 5:**
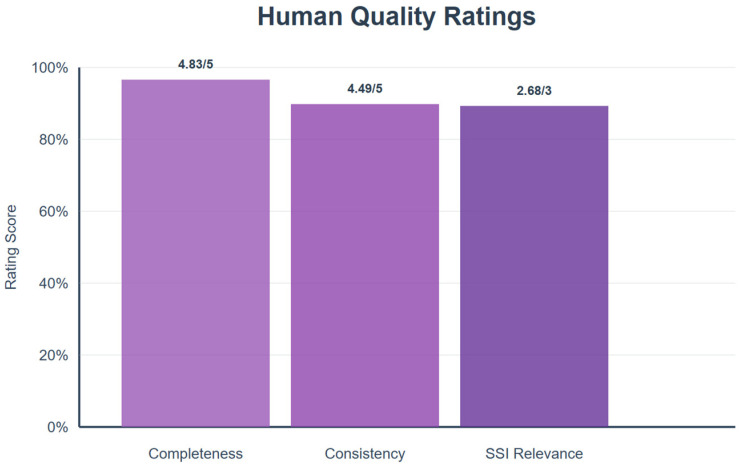
Manual evaluation results for completeness, relevance, and consistency. Data labels show mean reviewer scores: completeness = 4.83/5, consistency = 4.49/5, and relevance (SSI Index) = 2.68/3. These high scores indicate that system responses were consistently judged as comprehensive, relevant, and stable across paraphrased queries. Consistency was assessed across paraphrase variants (750 total queries).

Completeness, on a 5-point Likert scale, averaged 4.83, indicating comprehensive responses. Relevance assessed using the Sensibility–Specificity–Interestingness (SSI) Index (0–3 scale), averaged 2.68 out of 3. Consistency, assessed across paraphrase variants (750 total queries), averaged 4.49/5. Figure 5 presents these detailed human evaluation results.

#### 3.1.3. Inter-Rater Reliability

Inter-rater reliability analysis showed a Fleiss’ kappa of 0.609 (95% CI: 0.182–0.860), reflecting agreement beyond chance. All three reviewers agreed completely in 98% queries (245/250), while in the remaining 5 cases there was partial disagreement (2 vs. 1 split). Thus, all responses had a final consensus classification. The 5 cases with reviewer disagreement involved routine clinical scenarios where response adequacy required subjective judgment rather than clear-cut determinations. These disagreements reflected normal variation in clinical assessment rather than systematic bias or major safety concerns.

Pairwise Cohen’s kappa averaged 0.607 (range 0.437–0.722), and Krippendorff’s alpha for nominal accuracy was 0.609. Completeness ratings demonstrated strong ordinal reliability: ICC (2, k) 0.844 (95% CI: 0.81–0.87), ICC (2, 1) 0.643 (95% CI: 0.58–0.70), and Krippendorff’s alpha 0.675. However, consistency and SSI ratings showed lower inter-rater agreement (ICC < 0.40). Lower inter-rater reliability for subjective metrics in AI-generated medical responses stems from several factors. These include a lack of standardized rubrics, cognitive load from multi-dimensional evaluation, and the novelty of assessing conversational AI in healthcare. To address this, future work should focus on developing comprehensive scoring rubrics with clinical examples, structured rater training, Delphi methodology for consensus [48], and exploring sequential single-dimension rating. Mixed-methods approaches, like qualitative exit interviews, could also help understand scoring disagreements and refine evaluation frameworks.

#### 3.1.4. System Safety and Robustness (Human-Reviewed Aspects)

**Figure 6:**
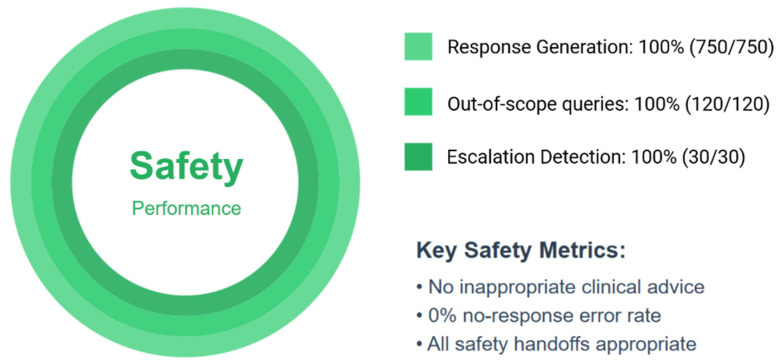
Safety and robustness performance of the AIVA Platform. System correctly identified 100% of out-of-scope queries (120 out of 120) and 100% of emergency escalation scenarios (30 out of 30), with no clinically unsafe responses generated. Created in BioRender. Haider, S. (2025) https://BioRender.com/zgs2hih.

The AIVA Platform correctly identified 100% of out-of-scope non-medical queries (*n* = 120/120), declining inappropriate clinical advice. All 100% emergency escalation scenarios were correctly flagged for urgent attention (*n* = 30/30) with appropriate safety handoff responses. The system produced valid responses for all 750 queries, resulting in a 0% no-response error rate. No clinically unsafe or inappropriate advice was generated in any evaluated scenario. All safety handoff responses for escalation scenarios were judged clinically appropriate by physician reviewers. Safety metrics are summarized in Figure 6.

### 3.2. Automated LLM Metrics Results

#### 3.2.1. Linguistic Fluency, Syntax and Readability

**Figure 7:**
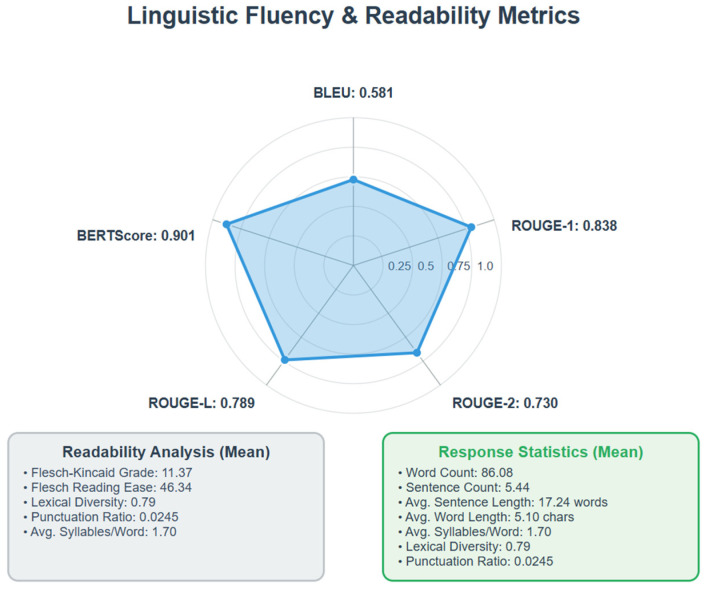
Automated evaluation of linguistic fluency and readability. Data labels show performance across metrics: BLEU = 0.58, ROUGE-1 = 0.84, ROUGE-2 = 0.73, ROUGE-L = 0.79, and BERTScore F1 = 0.90. Readability analysis indicated an average Flesch–Kincaid grade level of 11.4 and a Flesch Reading Ease score of 46.3, corresponding to an approximately 11th-grade reading level. These results demonstrate strong fluency and semantic alignment, though readability remains an area for optimization. Created in BioRender. Haider, S. (2025) https://BioRender.com/ifo4q9v.

The system achieved BLEU of 0.5814, ROUGE-1 0.8377, ROUGE-2 0.7302, ROUGE-L 0.7891, and BERTScore F1 0.9013. Responses averaged 86.08 words (5.44 sentences, 17.24 words/sentence), with an average word length of 5.10 characters, an average of 1.70 syllables/word, a lexical diversity of 0.79, and a punctuation ratio of 0.0245. Readability analysis indicated a Flesch–Kincaid Grade Level of 11.37 and a Flesch Reading Ease score of 46.34, corresponding to an approximately 11th-grade reading level. The distribution of responses by reading level across the 750 queries was as follows: 34.0% (255 responses) were at or below high school level, 48.3% (362 responses) were at a college level, and 17.7% (133 responses) were at a college graduate level. Figure 7 summarizes these linguistic fluency and readability metrics.

#### 3.2.2. Groundedness

**Figure 8:**
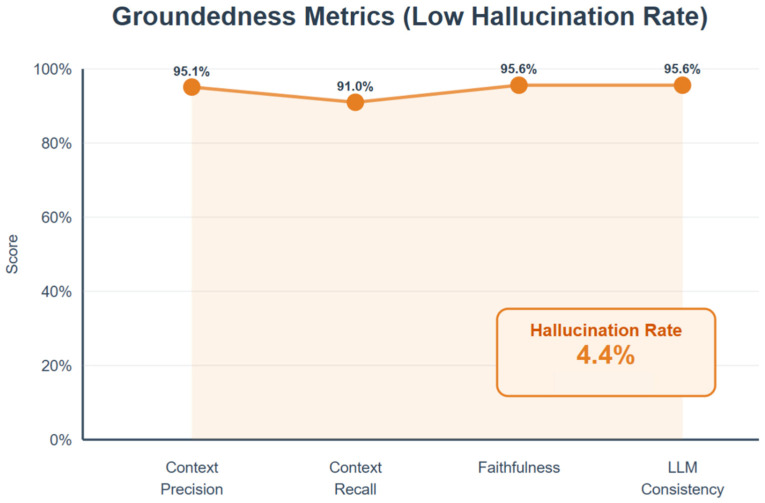
Groundedness metrics evaluating factual alignment of system responses. Data labels show context precision = 0.951, context recall = 0.910, and faithfulness = 0.956, corresponding to a hallucination rate of only 4.4%. Independent verification confirmed 95.6% agreement between generated answers and retrieved evidence. Created in BioRender. Haider, S. (2025) https://BioRender.com/3nnu90l.

The AIVA Platform achieved a context precision of 0.951, a context recall of 0.910, and a faithfulness of 0.956, indicating a hallucination rate of 4.4% across 750 interactions. Independent verification by Gemini 2.0 Flash further confirmed a 95.6% consistency between the retrieved evidence and the generated answers. Figure 8 provides groundedness metrics.

## 4. Discussion

This study demonstrates the AIVA Platform’s strong technical and clinical performance in addressing simulated postoperative care queries using RAG architecture. The system achieved an impressive overall accuracy of 98.4%, with perfect precision (1.0) and near-perfect recall (0.98). These robust classification metrics, achieved across a diverse corpus encompassing in-scope clinical questions, out-of-scope queries, and emergency escalation scenarios, underscore the platform’s high sensitivity in correctly answering clinical questions and excellent specificity in avoiding inappropriate or unsafe responses.

**Figure 9:**
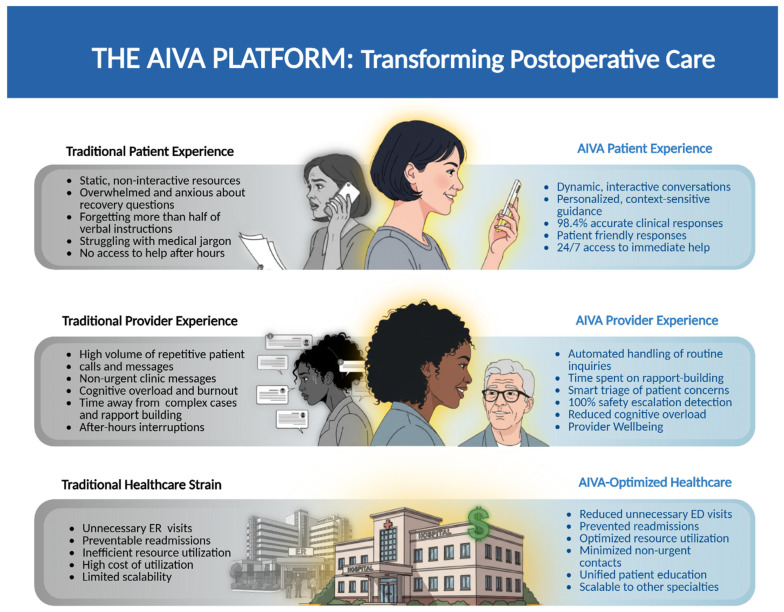
The AIVA Platform’s Transformative Potential. Created in BioRender. Haider, S. (2025) https://BioRender.com/zwhylxd.

The AIVA Platform provides clinical proof-of-concept for a safe and scalable RAG-based patient-facing postoperative education tool. By delivering interactive, conversational, evidence-based surgical recovery information, it directly addresses persistent information gaps post-discharge. In postoperative recovery, where patients often struggle with discharge instructions and caregivers bear significant responsibility for home care, such systems offer continuous, personalized guidance. Improved access to accurate, timely information can reduce postoperative complications arising from patient misunderstanding, nonadherence, or miscommunication. Concurrently, the system’s ability to address routine patient questions can reduce repetitive inquiries for surgical teams, thereby alleviating burnout and enabling them to focus on higher-acuity care (Figure 9).

Beyond immediate postoperative care, these findings lay the groundwork for integrating RAG conversational AI into broader digital health ecosystems. AIVA’s scalable architecture can integrate with other surgical and non-surgical specialties, remote monitoring, care coordination, and patient engagement tools, enhancing care continuity. Ultimately, such systems may reduce preventable readmissions, unnecessary emergency department (ED) visits, and non-urgent call volumes, improving patient outcomes and healthcare efficiency.

### 4.1. Advancement Beyond Earlier AIVA Iterations

The AIVA Platform demonstrates significant improvement over earlier intent-based AIVA iterations, which relied on rule-based NLP engines, such as Dialogflow, and pre-scripted responses. While accurate for narrowly defined intents, prior versions lacked adaptability for linguistic variability or unanticipated patient phrasing. By incorporating LLMs within a RAG architecture, the current system achieves greater linguistic flexibility for natural, conversational interactions while maintaining clinical safety.

The system expands clinical coverage from 10 to 20 high-frequency postoperative topics, broadening applicability. Enhanced safety features now detect out-of-scope queries, recognize escalation scenarios, and manage safety-critical conditions. Crucially, the RAG design grounds responses directly in a curated, physician-verified knowledge base, mitigating LLM hallucination risks while preserving conversational AI fluidity and accessibility.

### 4.2. Addressing LLM Safety Gaps: Retrieval-Augmented Generation as a Scalable Clinical Framework

In this section, we compare the AIVA Platform with earlier intent-based virtual assistants, commercial LLMs, and other recently developed RAG chatbots to highlight similarities and key distinctions.

Prior comparisons between earlier AIVA versions and commercial LLMs (e.g., ChatGPT-4, Bard) underscored the hallucination and factual inaccuracy risks of applying standalone generative models to clinical postoperative care [36,49]. In contrast, the AIVA Platform’s RAG design grounds responses in curated clinical evidence and applies robust safety filtering for out-of-scope and escalation scenarios, enabling clinically relevant and accurate outputs. This architecture provides a reproducible framework for specialty-specific assistants that adhere to emerging medical AI safety standards.

We observed four false negatives in our accuracy set, none of which represented unsafe or misleading content. These reflected incompleteness or scope mismatch relative to the gold standard (e.g., general advice without red-flag symptoms or partial wound-care guidance). Physician adjudication categorized all as low potential for harm, illustrating that while occasional incompleteness remains a limitation, the system did not produce unsafe guidance.

Across domains, RAG systems have consistently demonstrated high accuracy and reduced hallucination rates compared to baseline LLMs. For example, agentic frameworks for COVID-19 fact-checking achieved real-world accuracies above 0.95 (CRAG 0.972; SRAG 0.973) with strong contextual relevance [50]. Specialty-specific assistants like Thyro-GenAI achieved the highest response quality ranking among comparators (overall rank 3.0) [51], while LiVersa reached 100% binary accuracy in hepatology guideline questions but showed limitations in detailed rationale [52]. HandRAG reported strong correctness scores (0.79 average G-Eval) with semantic similarity of 0.75 [38]. Within this landscape, the AIVA Platform achieved 98.4% classification accuracy with perfect precision, 95.6% grounding verification consistency, and a low hallucination rate of 4.4%. AIVA is uniquely positioned as the first system to emphasize prospective safety validation in postoperative care, achieving a high success rate on its safety guardrails.

Agentic AI systems where a language model coordinates different specialized tools to solve complex problems are redefining how AI applications deliver domain-specific expertise. In healthcare, agentic AI is increasingly used to enhance both operational efficiency and clinical decision support, including symptom assessment, preliminary disease diagnosis, medication reminders, appointment scheduling, and personalized health information delivery [53,54,55]. In agricultural technology, multimodal large language models function as diagnostic agents, integrating image analysis, knowledge graphs, and retrieval systems to identify crop diseases and recommend pest management strategies [56,57]. While the AIVA Platform employs a streamlined RAG architecture rather than a fully agentic framework, it shares key design principles with these systems: grounding responses in curated evidence through retrieval augmentation and implementing safety guardrails for scope management.

Complementary reviews by Liu et al. [58], Miao et al. [59], Gargari et al. [60], confirm that RAG-augmented LLMs statistically outperform baseline models across bio-medical tasks. While study designs differ and direct head-to-head comparison is not possible, findings consistently show that RAG improves accuracy and grounding over standalone LLMs. Taken together, these findings situate the AIVA Platform at the upper range of reported RAG performance, while emphasizing its distinct contribution in surgical aftercare.

### 4.3. Readability Remains a Key Optimization Target

Despite AIVA’s high fluency and semantic fidelity, readability analysis suggests outputs may exceed ideal comprehension for many patient populations, averaging an estimated 11th-grade reading level (Flesch–Kincaid score of 46.3). This complexity can challenge patients with limited health literacy. Therefore, readability optimization is critical before large-scale clinical deployment. Strategies include readability-aware prompting to dynamically simplify responses, streamlining knowledge base authoring for plain language content, and post-processing language simplification modules. Future iterations of AIVA will incorporate readability-aware prompting and post-processing modules designed to target a 6th–8th grade reading level, with controlled fallbacks for clinically complex content. Additional strategies under development include teach-back style prompting, which encourages patients to confirm their understanding, and chunked action steps to simplify execution of postoperative care instructions. These approaches are crucial to ensure AI-generated guidance is both medically accurate and accessible.

### 4.4. Study Limitations

A primary limitation of this study is the use of simulated patient queries rather than real-world interactions. While in silico evaluations are a widely accepted first step in the validation of clinical AI systems, allowing controlled, reproducible, and risk-free testing—they cannot fully capture the complexity of real patient language, including ambiguity, spelling errors, dialectal variation, and diverse health literacy levels. Consequently, the clinical performance of the AIVA Platform in uncontrolled environments remains untested. As outlined in Future Directions (Section 4.6), we plan prospective, patient-facing trials to validate system performance in real-world surgical recovery settings. Second, lower inter-rater reliability for subjective metrics (e.g., consistency, relevance) highlights methodological challenges in evaluating AI-generated medical content. Third, the system was not subjected to formal adversarial or stress-testing with highly ambiguous queries.

Another limitation is that our supplementary “LLM-as-judge” groundedness check used the same model (Gemini 2.0 Flash) that generated the responses. While this secondary measure showed 95.6% agreement, future studies could strengthen validation by using different models for verification. Our primary evaluation framework relied on human expert reviewers and algorithmic groundedness metrics that are independent of the generative model. Future work for groundedness check will incorporate independent adjudicators and external models as evaluators to further minimize this risk. Finally, this evaluation focused exclusively on postoperative recovery content, limiting generalizability to other clinical domains without additional work.

### 4.5. Ethical Considerations

Despite strong performance, several unintended risks warrant careful consideration. Patient over trust in AI guidance could delay necessary clinical care, especially for subtle complications. Second, patient misinterpretation, even of accurate outputs, can occur due to varying health literacy or cognitive load during recovery. This underscores the importance of generating not only accurate content but also contextually accessible and easily understood outputs for diverse patient populations.

Future deployments require careful user interface design, explicit safety disclaimers, and user education to mitigate these risks. Patient-facing interfaces should clearly communicate system limitations, encourage appropriate use, and include embedded reminders to seek professional medical care. Incorporating periodic safety prompts, red-flag warnings, and symptom escalation triggers can further enhance safe system use. Patient and caregiver education on AI’s complementary role versus human clinical expertise will be critical for safe, effective integration.

### 4.6. Future Directions

Multiple efforts are advancing the AIVA Platform toward clinical translation within Mayo Clinic Cloud. As the next critical step, prospective real-world patient-facing pilot trials will be conducted to validate the system beyond simulation. These studies will evaluate usability, comprehension, safety, satisfaction, and clinical effectiveness in authentic patient–caregiver interactions, addressing the inherent limitations of in silico validation. Future research priorities also include rigorous adversarial and stress testing with highly ambiguous queries [61], integration with de-identified databases for enhanced personalization, real-time human-in-the-loop review [62], dedicated usability testing, and empathy model testing [63]. Broader work involves navigating, evolving regulatory and ethical frameworks and exploring integration with commercial communication platforms. Longitudinal evaluation will assess real-world impact on clinical outcomes, resource utilization, patient safety, and provider burden.

Future research should conduct systematic head-to-head comparisons of different foundation models (GPT-4, Claude, Llama, Gemini variants) implemented within identical RAG architectures and safety frameworks to determine which LLMs provide optimal performance for clinical virtual assistant applications while maintaining consistent evaluation conditions

The AIVA Platform’s RAG architecture offers multiple opportunities for broader clinical integration. It can serve as a first-line triage and education tool in remote monitoring programs [64], addressing routine questions while escalating concerning reports. Similarly, it can complement patient navigator programs by handling common inquiries, reducing human workload. Integration into care coordination platforms could enable continuous patient engagement, offering anticipatory guidance. Seamless embedding within digital health platforms would allow for scalable deployment while preserving clinician oversight, facilitating hybrid human–AI collaboration.

### 4.7. Generalizability to Other Clinical Domains

While this study focused on postoperative surgical care, AIVA Platform’s underlying architecture and safety framework are highly adaptable to other clinical domains with structured knowledge bases and ongoing patient self-management. Specialty areas like oncology, cardiology, endocrinology, chronic disease management, and rehabilitation could similarly benefit from scalable conversational AI support that provides evidence-based guidance with integrated safety guardrails and escalation protocols. This hybrid architecture combining retrieval grounding, domain-specific knowledge curation, and layered safety logic may serve as a generalizable template for developing future virtual assistants safely supporting patient care across diverse medical conditions.

## 5. Conclusions

The AIVA Platform demonstrates strong technical and clinical promise as a safe, accurate, and scalable postoperative patient education solution, due to its Retrieval-Augmented Generation architecture that effectively targets AI hallucination risks. In controlled evaluation, it achieved high accuracy in simulated queries, reflecting robust sensitivity and specificity, and its safety framework functioned as intended by effectively handling out-of-scope and emergency scenarios. While linguistic fluency and semantic fidelity were strong, the identified 11th-grade readability level highlights a key area for optimization. This work significantly contributes to emerging frameworks for safe, scalable, specialty-specific medical AI tools, effectively balancing dynamic, patient-centered conversational flexibility with strict clinical safety and content accuracy. These results form a strong foundation for prospective clinical trials and real-world deployment in surgical care settings.

## Figures and Tables

**Table 1:** Limitations of Current Patient Education and Gaps Addressed by the AIVA Platform.

Current Limitation	Impact on Patients/ Providers	Gap Addressed by AIVA
Static, text-heavy handouts/portals	Low engagement; poor comprehension	Interactive, conversational guidance
Complex medical jargon	Exceeds health literacy levels	Simplified, patient-friendly responses
Non-adaptable, generic content	Limited personalization	Context-specific, procedure-tailored answers
No interactivity or Q&A	Patient uncertainty, frequent calls	Dynamic query handling, immediate clarification
Limited access outside clinic hours	After-hours anxiety, ED overutilization	24/7 continuous availability

**Table 2:** Postoperative Care Knowledge Base Topics.

No.	Topic	No.	Topic
1	Pain and Pain Management	11	Showering and Bathing
2	Postoperative Nausea and Vomiting (PONV)	12	Emotional Wellbeing/Body image after surgery
3	Drain Management	13	Sleep Disturbance
4	Follow-up Appointments	14	Need for Home Assistance
5	Postoperative recovery and recovery timeline (fatigue, swelling, bruising)	15	Surgical Garments
6	Diet/Food to eat after surgery	16	Traveling
7	Resuming Physical activity (Gym, Weights)	17	Additional Treatments (Radiation/Chemo, etc.)
8	Scars	18	Alarm Signs
9	Sutures, Staples	19	Wound Care
10	Sexual Activity	20	Return to Work

**Table 3:** Test Corpus Composition.

Query Category	Base Queries (*n*)	+Paraphrase Variants (×2)	Total Queries (3 Variants: Base + Paraphrase)
In-Scope Clinical Queries	200	+400	600 (200 + 400)
Out-of-Scope Queries	40	+80	120 (40 + 80)
Escalation Scenarios	10	+20	30 (10 + 20)
**Total**	250	+500	**750 (500 + 250)**

**Table 4:** Evaluation Metrics and Sample Composition.

Metric	Queries (*n*)	Subset	Rules
Accuracy	250	Base queries only	Binary (0, 1)
Completeness	250	Base queries only	Likert (1–5)
Relevance (SSI)	250	Base queries only	SSI Scale (0–3)
Consistency	750	All queries (250 base + 500 paraphrased queries)	Likert (1–5)
Inter-rater Reliability	250	Base queries only	-

**Table 5:** Confusion Matrix for Accuracy.

	Predicted Positive	Predicted Negative
Actual Positive	196 (TP)	4 (FN)
Actual Negative	0 (FP)	50 (TN)

## Data Availability

The data collected for this study will be made available to others upon reasonable request. Specifically, the de-identified raw simulated query data and the curated, physician-verified knowledge base will be shared. Access to the data will be granted to qualified researchers for non-commercial research purposes upon submission of a formal request and execution of a signed data access agreement. Any additional restrictions or conditions for data use will be clearly outlined in the data access agreement.

## Supplementary Materials

The following supporting information can be downloaded at: https://www.mdpi.com/article/10.3390/bioengineering12111219/s1, Table S1: Representative annotation examples.
