# Supplementary material for: The Development and Evaluation of a Retrieval-Augmented Generation Large Language Model Virtual Assistant for Postoperative Instructions
Source: Bioengineering (Basel). 2025 Nov 7;12(11):1219. doi: 10.3390/bioengineering12111219 (PMC12649765; doi:10.3390/bioengineering12111219)
Supplement: Supplementary file 1 [file bioengineering-12-01219-s001.zip › bioengineering-3859990-supplementary.pdf]

Supplementary Table S1. Representative annotation examples

Sample queries, gold standard answers, AIVA Platform's outputs, and reviewer ratings. Reviewer scores (1 = correct, 0 = incorrect) were assigned independently by three blinded physicians. Final classification was determined by majority vote.

Queries	Gold Standard Answers	Simple question (Variant 1 Answers)	R1	R2	R3	Final Classification
**How much pain is normal after surgery?**	* It is normal to experience pain or discomfort after surgery.<br>* This is often referred to as acute post-surgical pain.<br>* Experiencing some level of discomfort is a normal part of the healing process.<br>* About 75% of patients report moderate to severe pain right after surgery.<br>* This pain typically lasts for days or weeks.<br>* Normal postoperative pain should gradually improve as healing progresses.	Feeling pain after surgery is common. After an operation, it is normal to experience acute post-surgical pain, typically lasting for days or weeks, improving as healing progresses. In fact, about 75% of patients report moderate to severe pain right after surgery. This acute pain should gradually improve as inflammation subsides. Is there anything else I can help you with today?	1	1	1	1
**When will my pain start to get better?**	* Acute post-surgical pain typically lasts for days or weeks.<br>* The pain should gradually improve as healing progresses.<br>* This improvement happens as inflammation subsides.<br>* Your pain should generally improve steadily and trend downward over time.<br>* Most pain will gradually improve over a few weeks as you heal.	Typically, surgical pain decreases significantly within a few weeks. Most pain after surgery will gradually improve over a few weeks as you heal. Is there anything else I can help you with today?	1	1	1	1
**What can I do besides medication to manage my pain?**	Besides medication, you can use non-drug techniques:<br>* Cold therapy (ice packs) to reduce pain and swelling.	There are many simple, non-drug techniques you can use to reduce pain. These methods can be used alongside medicines and tend to have minimal side	1	1	1	1

	* Elevating the affected limb to decrease swelling.<br>* Breathing and relaxation exercises (like deep breathing or listening to music) to calm your nervous system.<br>* Gentle movement (early mobilization) to improve circulation and reduce stiffness.<br>* Other options like TENS, massage, heat, or psychological support.<br><br>Combining these with medication is called multimodal analgesia and is very effective.	effects.<br><br>Here are several non-pharmacological pain management strategies commonly recommended after surgery:<br><br>Cold Therapy (Cryotherapy): Applying cold packs or ice to the surgical area can significantly reduce pain and swelling.<br>Other Modalities: There are additional non-drug methods that might be available depending on your resources and preferences, such as transcutaneous electrical nerve stimulation (TENS), massage therapy, heat therapy, and psychological support. Even simple distraction can help.<br>Is there anything else I can help you with today?					
**When should I call the doctor about nausea or vomiting?**	Call your doctor or seek care if you experience:<br><br>* Nausea or vomiting lasting longer than 24 hours.<br>* Inability to keep fluids down for more than 12 hours.<br>* Signs of dehydration (like dizziness, no urination, dry mouth).<br>* Bloody or coffee-ground vomit (go to ER).<br>* Severe abdominal pain or bloating.<br>* Severe headache or stiff neck with nausea.<br>* Fever, cough, or difficulty breathing after vomiting.<br>* Wound issues (pain, opening, drainage).	You should contact your doctor or seek medical care if you experience any of the following:<br><br>Nausea/vomiting lasting longer than 24 hours<br>Inability to keep fluids down<br>Signs of dehydration (very dark urine or no urine, a dry tongue or mouth, feeling lightheaded, or heart palpitations)<br>Vomiting blood or material that looks like "coffee grounds"<br>Severe abdominal pain or distension<br>Severe headache or stiff neck<br>High fever (above 101°F/38.5°C) with	1	1	1		1

	* If it's beyond what you can tolerate or not improving.	chills<br>Chest congestion, cough, or pain with breathing<br>Rapid or labored breathing<br>If you're vomiting everything you attempt to drink, you may need IV fluids and medications.	
